# Risk of depression mediates the association between cardiorespiratory fitness and academic performance in adolescent boys and girls: DADOS study

**DOI:** 10.1007/s00431-022-04645-y

**Published:** 2022-10-20

**Authors:** Irene Monzonís-Carda, Mireia Adelantado-Renau, Maria Reyes Beltran-Valls, Diego Moliner–Urdiales

**Affiliations:** LIFE Research Group, Faculty of Humanities and Social Sciences, Department of Education, Universitat Jaume I, Av. Vicent Sos Baynat, s/n, 12071 Castellon, Spain

**Keywords:** Mental health, Depression, Fitness, School performance, Adolescence, Moderated mediation

## Abstract

This study aims to analyze the mediating role of risk of depression in the association between cardiorespiratory fitness and academic performance in a sample of adolescents and to test the moderation effect of sex. A total of 263 adolescents (125 girls, 13.9 ± 0.3 years) from the DADOS (Deporte, ADOlescencia y Salud) study were included in the analysis. Cardiorespiratory fitness was assessed by the 20-m shuttle run test. Academic performance was assessed through the final academic grades and the test of educational abilities. Risk of depression was evaluated through the Behavior Assessment System for Children and Adolescents. Mediation analyses were performed to determine the direct and indirect associations between cardiorespiratory fitness, risk of depression, and academic performance. Indirect effects with confidence intervals not including zero were interpreted as statistically significant, and percentages of mediation were calculated in order to know how much of the association was explained by the mediation. Our findings indicated a significant mediating effect of risk of depression in the association between cardiorespiratory fitness with final grades in math, language, and grade point average (percentages of mediation: 26%, 53%, and 29%, respectively). These analyses were not moderated by sex (all confidence intervals included 0).

*Conclusion*: Risk of depression acts as a possible underlying mechanism in the association between cardiorespiratory fitness and academic grades in adolescents. Educational and health institutions could benefit from our findings since the promotion of higher cardiorespiratory fitness levels might reduce the risk of depression with potential benefits on adolescents’ academic performance.

**Table:** 

**What is Known:** *• Cardiorespiratory fitness is positively associated with academic performance in adolescents. Nevertheless, the psychological mechanisms underlying this association are poorly understood.*	
**What is New:** *• Risk of depression mediates the association between cardiorespiratory fitness and academic performance in adolescents, independently of sex.* • *Our findings may improve the efficacy of mental health and educational programs by promoting the enhancement of cardiorespiratory fitness levels, which may reduce risk of depression with potential benefits on academic performance. *

**What is Known:**

*• Cardiorespiratory fitness is positively associated with academic performance in adolescents. Nevertheless, the psychological mechanisms underlying this association are poorly understood.*

**What is New:**

*• Risk of depression mediates the association between cardiorespiratory fitness and academic performance in adolescents, independently of sex.*

• *Our findings may improve the efficacy of mental health and educational programs by promoting the enhancement of cardiorespiratory fitness levels, which may reduce risk of depression with potential benefits on academic performance. *

## Introduction

Mental health disorders are defined as the combination of the presence of psychological distress and the absence of psychological well-being [1]. The most common mental health disorder worldwide is depression [2] and its prevalence increases substantially during adolescence, particularly in girls [3]. Previous evidence suggests that the presence of common signs and symptoms of depression during this age span is expected to produce poor long-term outcomes, such as functional impairments and mental health difficulties [4]. Hence, strategies to reduce the onset of depressive symptoms in adolescents are needed.

Previous research in the adolescent population has shown that high levels of cardiorespiratory fitness (CRF), which is the ability of the circulatory and respiratory systems to supply oxygen when performing prolonged strenuous exercise [5], may protect against the development of depressive symptoms [6]. It is plausible that this effect is related to improvements in serotonin levels [7] and mood [8] derived from participation in regular physical activity, which is required to achieve high levels of CRF [5]. On the other hand, a growing body of evidence suggests that CRF is positively associated with brain structure and function in the preadolescent population, leading to better cognitive function and academic performance (AP) [9, 10].

AP refers to the educational goals achieved by students in a particular period of time and is usually assessed through academic grades or standardized tests [11]. Higher AP during adolescence has been associated with better health [12], especially in adolescent girls, which showed a 13% better future self-rated health status and a 1.4% lower body mass index for each standard deviation of AP. Moreover, higher AP has been also associated with better earnings in adulthood [13]. Thus, understanding the factors that influence AP in adolescents is essential for promoting future health and wealth.

The positive association between CRF and AP has been well-documented in our [14, 15] and other samples of adolescents [16]. Nevertheless, little is known about the mechanisms behind this association. Some of the explanations provided for this positive association are based on physical indicators, such as body mass index [15] and metabolic biomarkers, such as leptin or the brain-derived neurotrophic factor (BDNF) [14, 17]. In fact, higher fitness levels could be related to adolescents’ healthy weight status and optimal biomarkers concentration levels, which may be plausible explanations by which fitness may shape adolescents’ AP. On the other hand, neurobiological, psychosocial, and behavioral mechanisms have also been suggested to explain the positive association between CRF and AP [18]. Indeed, Lubans et al. [18] suggested that higher engagement in physical activity, as a proxy of fitness levels, may alter structural and functional compositions of the brain, provide improved self–perception and also confer better coping skills, which altogether may shape adolescents’ AP. Although multiple mechanisms might explain the association between CRF and AP, there is little evidence regarding common mental health disorders that usually emerge during the adolescence period [3].

To our knowledge, only one previous study has examined the mediating role of depressive symptoms in the association between physical fitness and AP in adolescents [19], suggesting that the combination of healthy fitness levels and lower depressive symptoms may lead to better AP. However, further research is needed in order to elucidate the interrelationship between CRF, depression, and AP during a phase of multiple physical, psychological, and emotional changes [20]. Therefore, the main aim of the current study was to analyze the mediating role of te risk of depression in the association between CRF and AP in a sample of adolescents. In addition, given the higher prevalence of risk of depression [3] and lower levels of CRF [21] among adolescent girls, we speculate that the mediation model presented above might be moderated by sex. Thus, a secondary aim was to investigate whether the mediating role of risk of depression in the association between CRF and AP was moderated by sex.

## Methods

### Study design and participants

The present study is part of the DADOS (Deporte, ADOlescencia y Salud) study, which is a 3-year longitudinal research project (from 2015 to 2017) aimed to investigate the influence of physical activity on health, cognition, and psychological well-being through adolescence. Advertising leaflets about the research project were sent to secondary schools and sports clubs located in Castellon province (Spain), which included main information about the aim, the study protocol, and the inclusion criteria: to be enrolled in second grade of secondary school (13–14 years old) and without diagnosed physical or neurological chronic diseases. Volunteers who met the inclusion criteria were included in the study. The results presented in this study belong to baseline data obtained between February and May of 2015. A total of 274 adolescents completed the baseline assessments of the DADOS study. From them, 4 participants were excluded due the lack of data on CRF, 1 participant was excluded because did not provide the official academic grades, 2 were excluded because did not report their pubertal stage, and 4 were excluded for not providing the information about their socioeconomic status (SES). Thus, a total of 263 adolescents (125 girls, 47.5%) were included in the analyses.

Adolescents and their parents or guardians were informed of the nature and characteristics of the study and all provided written informed consent. The DADOS study protocol was designed in accordance with the ethical guidelines of the Declaration of Helsinki 1964 (last revision of Fortaleza, Brazil, 2013) and approved by the Research Ethics Committee of Universitat Jaume I of Castellon (Spain).

### Cardiorespiratory fitness

CRF was assessed using the 20-m shuttle run test as described by Léger et al. [22]. Participants ran straight between 2 lines 20-m apart at a pace established by recorded audio signals. The initial speed was 8.5 km/h and it was increased by 0.5 km/h each minute. The test was completed when participants could not reach the end lines at the pace of the audio signals for 2 consecutive times or when they stopped because of fatigue. The last stage number completed was used to estimate the maximal oxygen uptake (VO_2max_, ml/kg/min) using the following equation reported by Léger et al. [22]: VO_2max_ = 31.025 + (3.238 × (8 + 0.5 × last stage number completed)) −(3.248 × age) + (0.1536 × (8 + 0.5 × last stage number completed) × age).

### Risk of depression

The S3 self-reported Spanish version of the Behavior Assessment System for Children and Adolescents (BASC-S3) questionnaire [23] was used to assess the risk of depression. BASC-S3 has been designed for adolescents aged 12–18 years old and consists of several statements that must be rated as true or false. Specifically, the scale of risk of depression is composed by 14 statements that assess adolescents’ feelings of loneliness, sadness, and their incapacity to enjoy life. Risk of depression score was calculated by transforming raw scores into standard T-scores with an average of 50 and standard deviations of 10 points. For the descriptive proposes of this study, the risk of depression score was dichotomized into “non-risk” (< 60) and “at risk” (≥ 60) according to the cut-off point established by Reynolds and Kamphaus [24]. The Spanish version of this questionnaire shows good internal consistency (Cronbach’s *α* = 0.81) [23]. In addition, optimal reliability results have also been obtained for the sample of the current study, showing high internal consistency (Cronbach’s *α* = 0.78).

### Academic performance

AP was assessed through two components: academic grades and academic abilities. First, we took the final academic grades from the first grade of secondary school, provided by each school’s secretary office. They were based on a ten-point scale (0 indicates the lowest achievement and 10 indicates the highest achievement). Individual grades for math, language, and grade point average (GPA) were included in the analyses. GPA score was defined as the average of the scores achieved by students in social sciences, natural sciences, math, language, and physical education. Second, we used the Spanish version of the Science Research Associates Test of Educational Ability (TEA) in order to measure academic abilities [25]. This test provides general measures of three areas of intelligence and skills of learning, which are obtained by adding the correct answers in each academic ability. Verbal ability evaluates the command of language (range: 0–50), numeric ability evaluates the speed and precision in performing operations with numbers and quantitative concepts (range: 0–30), and reasoning ability evaluates the skill to find logical order in sets of numbers, figures, or letters (range: 0–30). Based on the age range of our sample, level three of TEA questionnaire was used (internal consistency: verbal *α* = 0.74, numeric *α* = 0.87, and reasoning *α* = 0.77) [25].

### Covariates

Pubertal stage was self-reported using standardized pictures according to the five stages described by Tanner and Whitehouse [26], based on external primary and secondary sex characteristics. The degree of development was assessed through two components: pubic hair growth for girls and boys, in addition to breast development in girls and genital development in boys. A 5-point maturity rating (from 1 to 5) was used for each component, in which stage 1 corresponded to the prepubertal state and stage 5 to the mature state. The highest rating of the two components was used for data analyses.

SES was reported by the Family Affluence Scale, developed by Currie et al. [27]. It was used as a proxy of SES (ranging from 0 to 8), which is based on material conditions in the family such as car ownership, bedroom occupancy, computer ownership, and home internet access. For descriptive proposes, this variable was categorized into low (from 0 to 2), medium (from 3 to 5), and high (from 6 to 8) SES.

## Statistical analysis

Study sample characteristics are presented as mean ± standard deviation or frequencies and percentages for continuous and categorical variables, respectively. Differences between boys and girls were assessed using *t*-test for continuous variables and chi-square for categorical variables. Linear regression analyses were performed to confirm the relationships between CRF, risk of depression, and AP indicators; controlling for sex, pubertal stage, and SES.

**Fig. 1:**
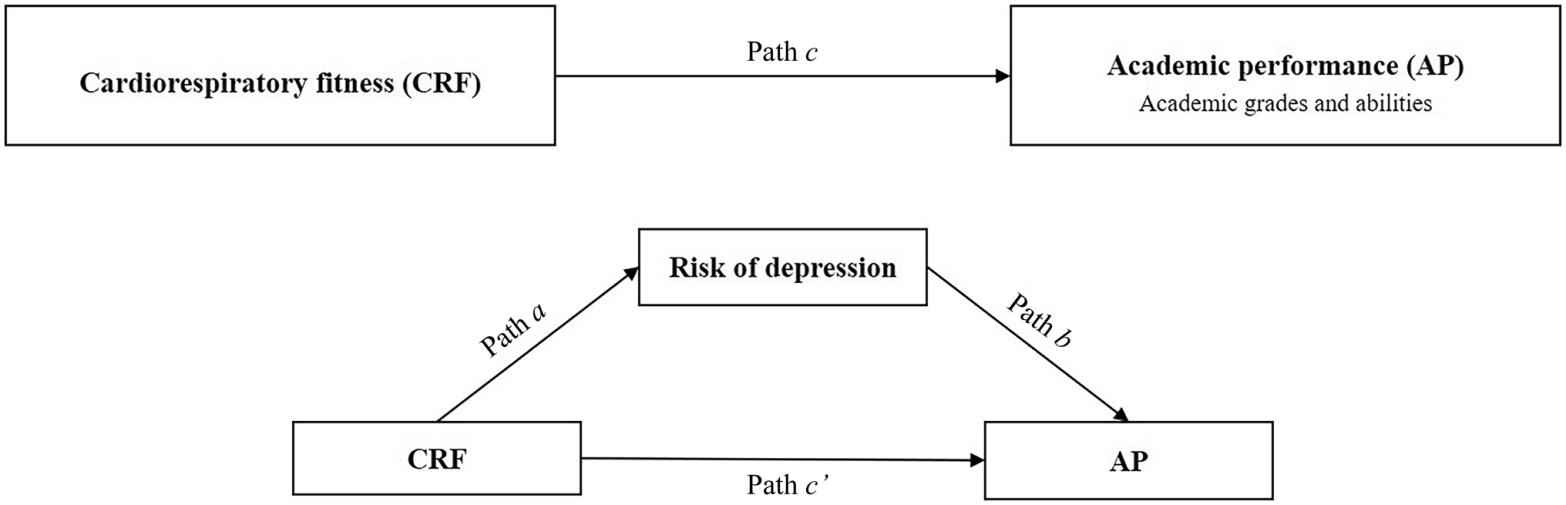
Schematic moderation model of risk of depression as the mediator between cardiorespiratory fitness (independent variable) and academic performance indicators (dependent variable) (Model 4)

**Fig. 2:**
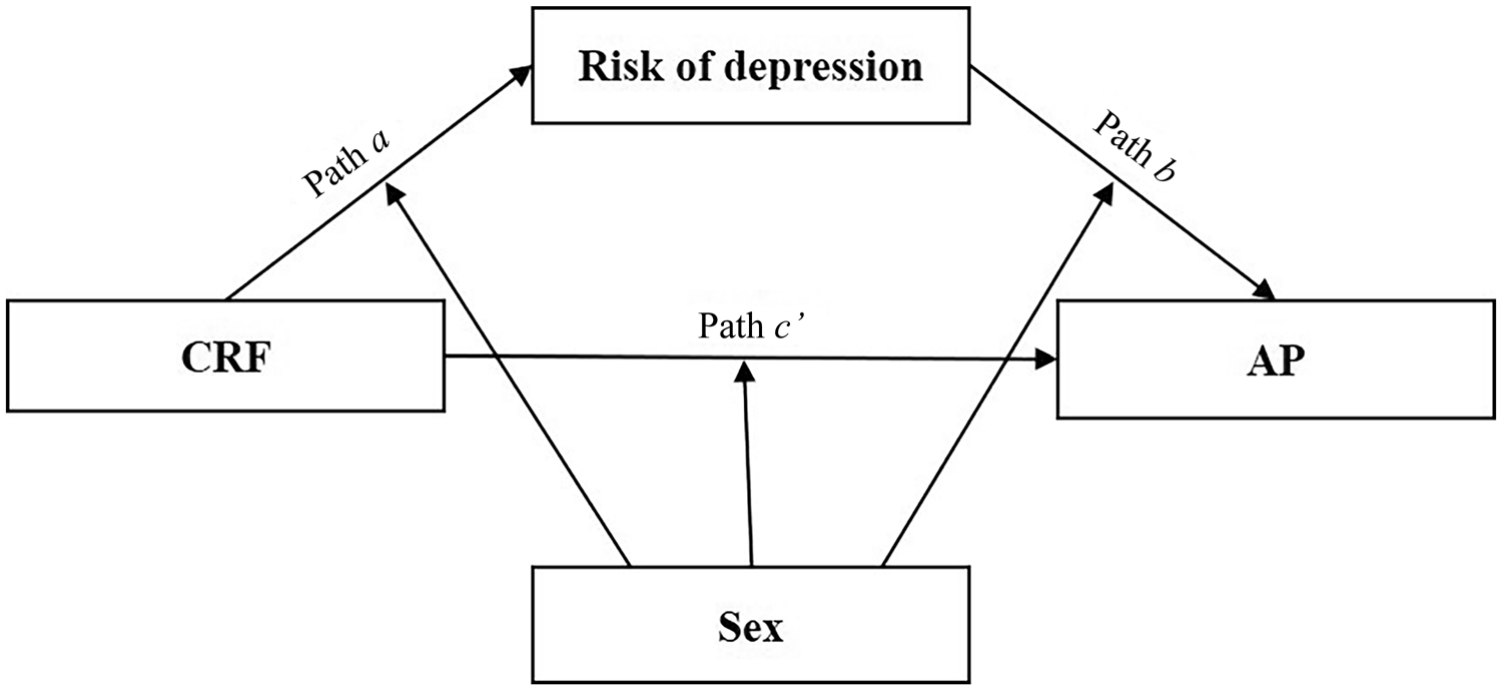
Schematic model of sex as a moderator of the mediation model (Model 59)

The mediation and moderated mediation models were analyzed using the PROCESS SPSS Macro version 2.16.3, with 5.000 bias-corrected bootstrap samples and 95% confidence intervals (CIs) [28]. Firstly, boot-strapped mediation procedures were performed to examine whether the association between CRF (independent variable) and AP (variables individually entered as the dependent variable) was mediated by the risk of depression score (mediator variable) using Model 4 (see Fig. 1). Indirect effects (paths a*b) with CIs not including zero were interpreted as statistically significant, which can be so regardless of the significance of the total effect (the effect of CRF on AP: path *c*) and the direct effect (the effect on AP when both CRF and risk of depression score were included as predictors: path *c’*) [28]. Percentage of mediation (P_M_) was calculated as [(indirect effect/total effect) × 100] in order to know how much of the total effect was explained by the mediation when the following assumptions were achieved: the total effect is larger than the indirect effect and with the same direction of the effect [28]. These analyses were adjusted by sex, pubertal stage, and SES. Next, Model 59 was used to test the moderated mediation effect, that was whether sex moderated the direct and indirect effects of CRF on AP (see Fig. 2). Likewise, if the 95% CI of the interaction did not contain 0, the moderated mediation effect could be interpreted as statistically significant. These analyses were adjusted by the pubertal stage and SES. All the analyses were performed using the IBM SPSS Statistics for Windows version 22.0 (Armonk, NY: IBM Corp) and the level of significance was set at *p* < 0.05.

## Results

The descriptive characteristics of the study sample are shown in Table 1. Overall, girls showed significantly lower CRF levels (54.0 vs 46.3, *p* < 0.001), higher SES (4.0 vs 4.4, *p* = 0.031), higher risk of depression score (44.9 vs 47.5, *p* = 0.011) and lower numeric ability than boys (14.7 vs 12.0, *p* < 0.001). In addition, the prevalence of participants at risk of depression was significantly higher in girls (2% vs 8%, *p* = 0.029).

**Table 1:** Descriptive characteristics of the study sample by sex

	All	Boys	Girls	*p*
	*N* = 263	*N* = 138	*N* = 125	
Physical characteristics				
Age (years)	13.9 ± 0.3	13.9 ± 0.3	13.9 ± 0.3	0.913
Pubertal stage (II–V) (%)	8/33/49/10	10/32/44/14	6/35/54/5	-
CRF (ml/kg/min)	50.3 ± 6.7	54.0 ± 5.3	46.3 ± 5.8	** < 0.001**
Socioeconomic status (0–8)	4.2 ± 1.4	4.0 ± 1.3	4.4 ± 1.4	**-**
Socioeconomic status (L/M/H) (%)	11/76/15	14/77/9	9/74/17	-
Risk of depression				
Risk of depression score (0–100)	46.2 ± 8.3	44.9 ± 6.0	47.5 ± 10.2	**0.011**
At risk of depression (%)	13 (4.9)	3 (2.2)	10 (8.0)	**0.029**
Academic performance				
Academic grades (0–10)				
Math	6.9 ± 1.6	7.0 ± 1.6	6.8 ± 1.6	0.281
Language	6.8 ± 1.5	6.6 ± 1.5	6.9 ± 1.5	0.147
Grade point average	7.1 ± 1.3	7.1 ± 1.3	7.2 ± 1.3	0.346
Academic abilities				
Verbal (0–50)	18.7 ± 5.3	19.1 ± 5.8	18.2 ± 4.6	0.171
Numeric (0–30)	13.4 ± 4.7	14.7 ± 4.5	12.0 ± 4.5	** < 0.001**
Reasoning (0–30)	16.5 ± 5.9	16.1 ± 5.7	17.0 ± 6.1	0.214

Values are mean ± standard deviation or frequency (%). Differences between sex were examined by t-test or chi-square test. Values in bold indicate significant results

*CRF* Cardiorespiratory Fitness, *L* Low Socioeconomic Status, *M* Medium Socioeconomic Status, *H* High Socioeconomic Status

Linear regressions between CRF, risk of depression score, and AP indicators, controlling for sex, pubertal stage, and SES are shown in Table 2. Overall, CRF was positively associated with math (*β* = 0.173, *p* = 0.021), GPA (*β* = 0.169, *p* = 0.024) and numeric ability (*β* = 0.167, *p* = 0.021), whereas risk of depression score was negatively associated with CRF (*β* =  −0.230, *p* = 0.002) and all academic grades indicators (*β* ranging from −0.262 to −0.216, all *p* < 0.001).

**Table 2:** Linear regression analyses between cardiorespiratory fitness, risk of depression score, and academic performance indicators in adolescents

	**CRF**			**Risk of depression score**	
	*ꞵ*	*p*		*ꞵ*	*p*
CRF	−	−		− 0.230	**0.002**
Academic performance					
Academic grades					
Math	0.173	**0.021**		− 0.216	**< 0.001**
Language	0.111	0.141		−0.262	**< 0.001**
Grade point average	0.169	0.024		−0.231	**< 0.001**
Academic abilities					
Verbal	0.117	0.120		−0.053	0.393
Numeric	0.167	**0.021**		−0.086	0.153
Reasoning	0.029	0.701		−0.036	0.563

Analyses were adjusted by sex, pubertal stage, and socioeconomic status. Values in bold indicate statistically significant results

ꞵ standardized regression coefficient, *CRF* Cardiorespiratory Fitness

Simple mediation analyses results are presented in Table 3, which show that CRF indirectly influenced some AP indicators through the risk of depression score, controlling for sex, pubertal stage, and SES. Specifically, CRF was negatively associated with the risk of depression score (path *a*), and the risk of depression score was negatively associated with academic grades (path *b*). Moreover, a significant total relationship was observed for CRF with math, GPA, and numeric ability (path *c*). The direct association between CRF and AP, when the risk of depression score was included in the model as a covariate (path *c’*), was significant for numeric ability. Regarding the indirect association (path a*b), the results showed that the risk of depression mediates the association of CRF with all final academic grades, but not with academic abilities. Specifically, our results suggest that high CRF levels may reduce the risk of depression, which in turn, may contribute to better academic grades in math, language, and GPA (with a P_M_ ranging from 26 to 53%).

**Table 3:** Total, direct, and indirect effects, *a* and *b* pathways, of the mediation analyses investigating risk of depression as a mediator between cardiorespiratory fitness and academic performance indicators in adolescents (Model 4)

	Total effect (path *c*)			Direct effect (path *c’*)			Path *a*			Path *b*			Indirect effect (path a*b)							P_M (%)_
	B	SE	p	B	SE	p	B	SE	p	B	SE	p	B	SE	LLCI			ULCI		
Academic grades																				
Math	0.041	(0.018)	**0.021**	0.030	(0.018)	0.088	−0.285	(0.091)	**0.002**	−0.037	(0.012)	**0.002**	**0.011**	**(0.004)**	0.004		0.020			26
Language	0.025	(0.017)	0.141	0.012	(0.017)	0.478	−0.285	(0.091)	**0.002**	−0.047	(0.011)	**0.000**	**0.013**	**(0.005)**	0.004		0.024			53
Grade point average	0.032	(0.014)	**0.024**	0.023	(0.014)	0.108	−0.285	(0.091)	**0.002**	−0.033	(0.010)	**0.001**	**0.009**	**(0.004)**	0.003		0.017			29
Academic abilities																				
Verbal	0.092	(0.059)	0.120	0.085	(0.060)	0.157	−0.285	(0.091)	**0.002**	−0.023	(0.040)	0.568	0.007	(0.010)	−0.013		0.027			-
Numeric	0.117	(0.050)	**0.021**	0.107	(0.051)	**0.038**	−0.285	(0.091)	**0.002**	−0.035	(0.034)	0.310	0.010	(0.008)	−0.005		0.027			-
Reasoning	0.025	(0.066)	0.701	0.019	(0.067)	0.781	−0.285	(0.091)	**0.002**	0.023	(0.045)	0.608	0.007	(0.014)	−0.026		0.031			-

Results showed as unstandardized coefficients (standard error and bias-corrected 95% confidence interval based on 5000 bootstraps). All analyses were adjusted for sex, pubertal stage, and socioeconomic status. Values in bold indicate significant results. Particularly, the indirect effect (path a*b) indicates significant results when 0 is not in the 95% confidence interval

*B* unstandardized coefficient, *SE* standard error, *LLCI* lower level of confidence interval, *ULCI* Upper Level of Confidence Interval, *P*_*M*_ Percentage of Mediation

Table 4 presents the results of the individual paths from the moderated mediation analyses. These results showed that sex only played a moderating role in the association between risk of depression and reasoning ability when adjusting for CRF (path *b*: *B* =  −0.201, *p* = 0.045, 95% CI: −0.397, −0.005), showing a trend in boys but not in girls (boys: *B* =  −0.163, *p* = 0.053 vs girls: *B* = 0.038, *p* = 0.480; data not shown). However, sex did not moderate any other association (path *a*: CRF—risk of depression; path *b*: risk of depression—math, language, GPA, verbal ability, and numeric ability with CRF included as a covariate) neither the direct association between CRF and AP when risk of depression score was included as a covariate (path *c’*) (all 95% CIs included 0).

**Table 4:** Moderated mediation analysis (Model 59)

Variable		B		SE	*t*		p	LLCI		ULCI	
Outcome: risk of depression											
CRF	−		0.448	0.127	−	3.520	**0.001**	−	**0.699**	−	**0.197**
Sex	−		17.329	9.345	−	1.854	0.065	−	35.732		1.074
CRF*sex			0.338	0.184		1.831	0.068	−	0.025		0.701
Outcome: math											
CRF			0.021	0.025	−	0.822	0.412	−	0.029		0.070
Risk of depression	−		0.036	0.014	−	2.581	**0.010**	−	**0.064**	−	**0.009**
Sex	−		0.760	2.303	−	0.330	0.742	−	5.294		3.775
CRF*sex			0.019	0.036		0.542	0.588	−	0.051		0.090
Risk of depression*sex	−		0.006	0.026	−	0.222	0.824	−	0.058		0.046
Outcome: language											
CRF			0.007	0.024		0.297	0.767	−	0.040		0.055
Risk of depression	−		0.036	0.014	−	2.629	**0.009**	−	**0.062**	−	**0.009**
Sex	−		0.799	2.212	−	0.361	0.718	−	3.557		5.156
CRF*sex			0.012	0.034		0.346	0.729	−	0.056		0.079
Risk of depression*sex	−		0.041	0.025	−	1.618	0.107	−	0.091		0.009
Outcome: GPA											
CRF			0.024	0.020		1.191	0.235	−	0.016		0.064
Risk of depression	−		0.028	0.011	−	2.419	**0.016**	−	**0.050**	−	**0.005**
Sex			0.512	1.865		0.275	0.784	−	3.161		4.185
CRF*sex	−		0.002	0.029	−	0.054	0.957	−	0.059		0.055
Risk of depression*sex	−		0.018	0.021	−	0.826	0.410	−	0.060		0.024
Outcome: verbal ability											
CRF			0.123	0.085		1.439	0.152	−	0.045		0.291
Risk of depression	−		0.026	0.048	−	0.529	0.597	−	0.120		0.069
Sex			3.188	7.864		0.405	0.686	−	12.298		18.674
CRF*sex	−		0.078	0.122	−	0.636	0.525	−	0.318		0.163
Risk of depression*sex			0.020	0.090		0.217	0.828	−	0.157		0.196
Outcome: numeric ability											
CRF			0.142	0.073		1.945	0.053	−	0.002		0.286
Risk of depression	−		0.015	0.041	−	0.352	0.726	−	0.096		0.067
Sex			8.190	6.717		1.219	0.224	−	5.037		21.417
CRF*sex	−		0.068	0.104	−	0.648	0.517	−	0.273		0.138
Risk of depression*sex	−		0.064	0.077	−	0.828	0.408	−	0.215		0.088
Outcome: reasoning ability											
CRF			0.078	0.095		0.825	0.410	−	0.109		0.265
Risk of depression			0.038	0.054		0.707	0.480	−	0.068		0.143
Sex			13.651	8.732		1.563	0.119	−	3.546		30.848
CRF*sex	−		0.110	0.135	−	0.814	0.416	−	0.377		0.156
Risk of depression*sex	−		0.201	0.100	−	2.017	**0.045**	−	**0.397**	−	**0.005**

All analyses were adjusted for the pubertal stage and socioeconomic status. Statistically significant effects indicating that 0 is not in the 95% confidence interval of the indirect effect are presented in bold

*B*, unstandardized coefficient, *SE* Standard Error, *LLCI* Lower Level of Confidence Interval, *ULCI* Upper Level of Confidence Interval, *CRF* Cardiorespiratory Fitness, *GPA* Grade Point Average

^*^p < 0.05; ***p < 0.001

The moderated mediation model was further tested by analyzing the association between CRF on AP through the risk of depression at the different conditions of the moderator (i.e., girls and boys). As shown in Table 5, risk of depression significantly mediated the association between CRF and AP in girls when the dependent variables were math (*B* = 0.016, 95% CI: 0.004, 0.034), language (*B* = 0.016, 95% CI: 0.001, 0.035), and GPA (*B* = 0.012, 95% CI: 0.002, 0.027). Nevertheless, the results indicated that sex did not significantly moderate this mediation model (all 95% CIs included 0).

**Table 5:** Conditional indirect effects of cardiorespiratory fitness on academic performance at the conditions of the moderator (Model 59)

	B		SE	LLCI		ULCI	Index [LLCI; ULCI]
Outcome: math							
Girls		0.016	0.008		**0.004**	**0.034**	0.012 [−0.030; 0.005]
Boys		0.005	0.005	−	0.002	0.016	
Outcome: language							
Girls		0.016	0.009		**0.001**	**0.035**	0.008 [-0.030; 0.012]
Boys		0.085	0.006	−	0.004	0.022	
Outcome: GPA							
Girls		0.012	0.007		**0.002**	**0.027**	0.012 [−0.024; 0.008]
Boys		0.005	0.005	−	0.002	0.016	
Outcome: verbal ability							
Girls		0.011	0.016	−	0.024	0.043	0.011 [−0.047; 0.035]
Boys		0.001	0.012	−	0.020	0.031	
Outcome: numeric ability							
Girls		0.007	0.151	−	0.023	0.040	0.002 [−0.034; 0.034]
Boys		0.009	0.008	−	0.007	0.026	
Outcome variable: reasoning ability							
Girls	−	0.017	0.032	−	0.092	0.034	0.035 [−0.023; 0.118]
Boys		0.018	0.016	−	0.008	0.055	

All analyses were adjusted for the pubertal stage and socioeconomic status. Statistically significant effects indicating that 0 is not in the 95% confidence interval of the indirect effect are presented in bold

*B* unstandardized coefficient, *SE* Standard Error, *LLCI* Lower Level of Confidence Interval, *ULCI* Upper Level of Confidence Interval, *GPA* Grade Point Average

## Discussion

The main findings of the present study indicated that the risk of depression mediated the positive association between CRF and AP in adolescents. Moreover, our results revealed that sex did not act as a moderator on this mediation model. These results contribute to the current scientific knowledge by suggesting the key role of risk of depression in the association between CRF and AP, independently of sex.

Since mediation analysis assumes that the independent variable has an effect on the mediator, influencing the dependent variable, our results suggest that high CRF levels may reduce the risk of depression, which in turn, may contribute to better AP. These findings are consistent with the only previous study in which depression showed to be an important factor driving the association of CRF and AP in a sample of 144 adolescents, including health-related and skill-related physical fitness [19]. Additionally, our results agree with previous studies showing inverse associations between CRF and depressive symptoms [29], as well as between the early identification of depression and AP among adolescents [30].

The positive association between CRF and AP explained by the risk of depression may be related to both psychological and physiological mechanisms. On the one hand, prior research in the adolescent population has shown that higher CRF is positively associated with better mood, self-esteem, and social support among equals, which may provide a sense of psychological well-being [31–33], diminishing depressive symptomatology. In fact, adolescents without depressive symptoms have a higher interest in school activities and an increased ability to think or concentrate than their depressed peers [34]. Thus, we speculate that these changes on depressive symptoms could shape adolescents’ AP [30]. On the other hand, CRF may optimize leptin concentration and central levels of BDNF, which are metabolic biomarkers involved in emotional and neurocognitive processes [17, 35]. Reduced leptin concentration and increased BDNF levels in physically fit adolescents are related to lower levels of depression and have also been positively associated with memory function [32, 35, 36], which may be related to lower depressive symptoms and cognition, positively influencing AP. Taken together, the previous evidence supports our data suggesting that higher levels of CRF may have beneficial effects in reducing the risk of developing depression, leading to higher AP in the adolescent population.

The moderated mediation analyses revealed that sex did not moderate the association between CRF and AP mediated by the risk of depression. To our knowledge, this is the first study investigating this moderated mediation model, which hampers direct comparisons. Yet, our results partially concur with previous studies showing that the positive association between CRF and AP [37], the negative association between CRF and depression [6], as well as the negative association between depression and AP [30] were not dependent on sex. However, despite these results support our findings, some previous evidence has suggested significant results only for adolescent boys or girls. Indeed, Castelli et al. [37] suggested that the positive association between CRF and AP was significant only in adolescent girls, while Álvarez-Bueno et al. [16] showed a stronger significant association in boys than in girls. In addition, Ruggero et al. [29] suggested that higher CRF levels were associated with fewer depressive symptoms among adolescent girls, while Åvitsland et al. [38] found a significant association among boys and only in girls with high SES. Likewise, Needham et al. [39] found a significant association between depressive symptoms and AP in adolescent girls, while Obradovic et al. [40] found this significant association only among adolescent boys. Despite the existing divergent results regarding the interaction of sex on the investigated associations, our findings provide a new insight for considering the adolescence population altogether when designing programs aimed to improve physical and mental health, as well as AP.

Mediation and moderated mediation analyses only showed significant results when considering academic grades as the dependent variable, but not when considering academic abilities. These divergent results for AP variables may be explained by the fact that academic grades and abilities rely on different forms of assessment. Academic grades are the result of the student progression during an academic year, while academic abilities are assessed by a standardized test in a single time-point trial [41]. Additionally, unlike academic abilities’ test, which mainly requires good cognitive skills, the multidimensional nature of academic grades could also involve the teachers perceptions of secondary school students’ prosocial behavior [42].

## Limitations and strengths

The results of the current study should be interpreted with caution. First, the cross-sectional design of our analyses prevents us from interfering causal relationships. Second, biomarkers closely linked to VO_2max_ trainability and depression, such as genetics [43, 44], were not considered. Third, the risk of depression, pubertal stage, and SES were self-reported and may limit the participants to certain answer choices. Lastly, the use of a standardized test of academic abilities and the official academic grades, although socially valid measures of AP, can be subject to biases. Nonetheless, our mediation analysis strategy allowed us to provide novel data supporting the importance of the risk of depression in the CRF and AP association. Moreover, the moderated mediation analysis used is a robust statistical measure for exploring the moderation effect of sex. Additional strengths comprise the use of objective and standardized measures of CRF, AP indicators, as well as a relatively large and age-matched sample of adolescents. Alongside these considerations, as suggested by prior research [11, 45, 46], our statistical analyses were controlled for the pubertal stage and SES, which are relevant given their associations with CRF, depression, and AP.

## Conclusion

The current study showed that the risk of depression plays a mediating role in the positive association between CRF and final academic grades in math, language, and GPA in adolescents, independently of sex. Thus, our data suggest that enhancing CRF during adolescence through healthy lifestyle promotion may contribute to better AP, partially via reductions in the risk of developing depression. Our findings may have significant implications for public health and education professionals, suggesting that programs addressing both physical and mental health issues may benefit adolescents’ AP. Further larger longitudinal and interventional studies are warranted to elucidate the pathways by which CRF levels may diminish the risk of depression, shaping the AP in the adolescent population.

## Data Availability

Not applicable.

## Abbreviations

AP: Academic performance
CIs: Confidence intervals
CRF: Cardiorespiratory fitness
GPA: Grade point average
SES: Socioeconomic status
